# Advanced Nanovehicles-Enabled Delivery Systems of Epigallocatechin Gallate for Cancer Therapy

**DOI:** 10.3389/fchem.2020.573297

**Published:** 2020-10-23

**Authors:** Kai Li, Chao Teng, Qianhao Min

**Affiliations:** ^1^Shenzhen Polytechnic, Institute of Marine Biomedicine, Shenzhen, China; ^2^State Key Laboratory of Analytical Chemistry for Life Science, School of Chemistry and Chemical Engineering, Nanjing University, Nanjing, China

**Keywords:** EGCG, cancer, nanovehicles, drug delivery systems, combination therapy

## Abstract

Epigallocatechin gallate (EGCG) is the most abundant polyphenolic constituent derived from green tea extract, which has demonstrated versatile bioactivities in combating cardiovascular diseases, neurodegenerative diseases, diabetes, and cancer. In light of its anticancer activity, increasing attention has been paid to developing potent strategies involving EGCG in cancer chemotherapy. However, the poor bioavailability and stability of EGCG limits its effectiveness and practicality in real biomedical applications. To overcome this drawback, nanotechnology-facilitated drug delivery systems have been introduced and intensively explored to enhance the bioavailability and therapeutic efficacy of EGCG in cancer treatments and interventions. This review briefly discusses the anticancer mechanisms of EGCG, and then summarizes recent advances in engineering nanovehicles for encapsulating and delivering EGCG toward cancer therapy. In addition, we also highlight successful integrations of EGCG delivery with other chemotherapies, gene therapies, and phototherapies in one nanostructured entity for a combination therapy of cancers. To conclude, the current challenges and future prospects of the nanovehicle-based transportation systems of EGCG for cancer therapy are also discussed.

## Introduction

**Figure 1:**
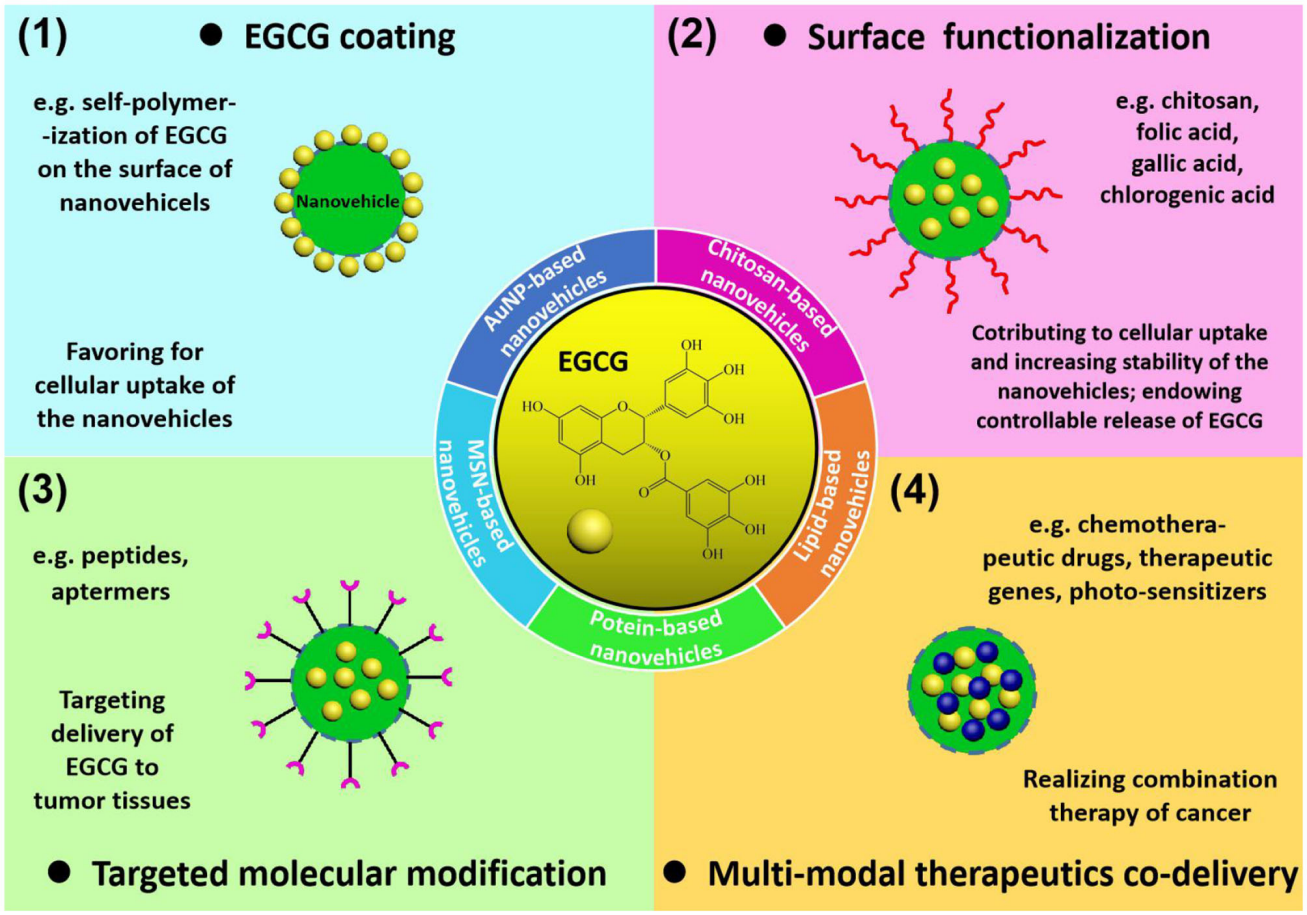
Summary of EGCG delivery systems: (1) grafting EGCG on the surface of the nanoparticles to enhance cellular uptake of as-obtained nanovehicles; (2) surface functionalization with specific molecules (chitosan, folic acid, gallic acid, and chlorogenic acid) to enhance stability, cellular uptake, and drug release properties of the nanovehicles; (3) targeted molecular modification by peptides or aptamers to target specific cancer cell receptors; (4) construction of multi-modal therapeutics co-delivery systems to realize EGCG-involved cancer combination therapy.

Natural products hold great potential in the fields of biomedical research, drug development, and clinical application, as they can serve as medicinal sources for the treatment of cancer, bacterial and fungal infections, inflammation, and other diseases (Mignani et al., 2018). Particularly, the superior biocompatibility, broad spectrum of biological activity, and specifically targeted effects of these compounds make them potent cancer chemoprevention and chemotherapy agents with minimal side effects (Cragg et al., 2009; Spradlin et al., 2019). Moreover, these abundant and widely varied sources can reduce the cost of cancer treatment. Epigallocatechin gallate (EGCG) is known as the most biologically active catechin derived from green tea extracts. Benefiting from the eight free hydroxyl groups in its flavone-3-ol phenolic structure (Figure 1), EGCG demonstrates unique merits in free radical scavenging, accounting for its biological functions in biomedicine (antioxidation, anti-inflammatory, reduction of blood lipids, and sugar, etc.) (Cai et al., 2006; Chakrawarti et al., 2016). Since the recognition of its anticancer effects, EGCG has in recent years been intensively investigated as a star phytochemical for regulating or inhibiting the physiological changes during canceration (Singh et al., 2011; Granja et al., 2016; Gan et al., 2017).

According to previous literature, the anticancer mechanism of EGCG involves many pathways. Specifically, the tumor suppressive behavior of EGCG through pro-oxidative effects is based on covalent binding of EGCG with antioxidants such as glutathione (GS), then producing a certain amount of reactive oxygen species (ROS) to activate the pro-oxidative signaling pathways (Shankar et al., 2012) and induce apoptosis of tumor cells (Li et al., 2010). EGCG can also target a variety of cancer-related membrane receptors (Singh and Katiyar, 2013) or nucleus signaling receptors (Shankar et al., 2012) and down regulate or inhibit their abnormal expression, therefore inhibiting the proliferation and metastasis of tumor cells. Evidence also shows that EGCG modulates signaling cascades to induce programmed cell death through activating protein kinases (Zhu et al., 2017). Additionally, suppression of vascular endothelial growth factor (VEGF) (Wang J. et al., 2018), eliminations of cancer stem cell (CSC)-characteristics (Xia and Xu, 2015), and down-regulation of telomerase (Sadava et al., 2007; Berletch et al., 2008) have been proposed to be involved in the curative mechanisms of EGCG.

In view of its anticancer activity, EGCG has been exploited as a potent chemotherapeutic agent for various cancers. However, the poor bioavailability and stability of EGCG restricts its clinical applications. As shown by Nakagawa and Miyazawa (1997) the maximum concentration of EGCG is calculated as 0.32% of ingested EGCG in human plasma through oral administration, which is too low to exhibit ideal efficacy. The methylation, glucuronidation, sulfation, and oxidative degradation of EGCG during its metabolic process further weakened its efficacy. Therefore, it is essential to develop novel drug delivery systems that enhance the stability and bioavailability of EGCG, thus ensuring the effective concentration and bioactivity *in vivo* (Yang et al., 2019). As the use of nanotechnology-facilitated biomedicine in the past decades has flourished, encapsulating EGCG within nanovehicles has been adopted by many researchers (Granja et al., 2016; Ye and Augustin, 2018). The protection imparted by nanovehicles allowed the isolation of entrapped EGCG from the outer physiological conditions, thus avoiding the probable compositional and structural change of the bioactive chemical cargo. Further, nanovehicles featuring high drug loading, targeted drug transportation, and site-specific drug release significantly improved the bioavailability of EGCG with sufficient concentrations in the lesions, making the precise treatment of cancer possible. To further augment the therapeutic outcomes, the combination of EGCG-dominated chemotherapy with other chemicals (Hu et al., 2015; Zhou et al., 2016), therapeutic genes (Ding et al., 2018; Liang et al., 2018), and photothermal agents or photosensitizers (Mun et al., 2014; Qi et al., 2014) has received numerous successes in boosting efficacy and minimizing drawbacks in cancer treatments. This article mainly focuses on recent advances in engineering nanovehicles for EGCG encapsulation and delivery for precise and efficient cancer therapy. Moreover, endeavors devoted to integrating EGCG-involved chemotherapy with other chemical curative agents, therapeutic genes, and phototherapeutic components in a single nanovehicle for elevating the overall potency are also overviewed.

## EGCG Delivery Systems Enabled by Nanovehicles for Cancer Therapy

The construction of EGCG-loaded nanovehicles has been generally recognized to enhance the stability and bioavailability of EGCG, exhibiting great potential for practical applications in cancer chemoprevention and chemotherapy. Numerous nanomaterials, including gold nanoparticles, mesoporous silica nanostructures, chitosan nanoparticles, lipid nanoparticles, and protein nanoassemblies, can serve as carriers for delivering EGCG to tumor tissues, while the delivery efficiency is highly dependent on the morphological and surface characteristics of nanovehicles (Figure 1). The physiological stability and cellular uptake of nanovehicles in tumor sites relies on the composition, dimension, outer surface chemistry, and electrostatics of the drug-loaded nanoparticles. In addition, targeted delivery and controllable release of the entrapped therapeutic agents is determined by engineering of nanovehicles with recognition ligands and bioresponsive species. In this section, the nanovehicles for EGCG transportation differing in building components, surface functionalities, drug release mechanisms, and biomedical uses are elucidated in detail. The main characteristics of nanostructure-based EGCG delivery systems for cancer treatments are summarized in Table 1.

**Table 1:** Summary of gold-, mesoporous silica-, lipid-, chitosan-, and protein-based EGCG delivery systems.

**EGCG nano- delivery system**	**Synthetic method**	**Particle size (nm)**	**Encapsulation efficiency (%)**	**Release of EGCG**	**Cell lines/animal models**	**Achievement**	**References**
**Gold nanoparticles-based EGCG delivery systems**							
EGCG-AuNPs	Reduction of sodium tetrachloro aurate by EGCG	20–40	—	—	PC-3 cells	EGCG-AuNPs internalize selectively within PC3 cells providing threshold concentrations required for photoacoustic signals.	Viator et al., 2010
EGCG-gold nanoparticles (E-GNPs)	Reduction of HAuCl_4_·3H_2_O by EGCG	25	0.235 (w/w)	42.9% (37°C, 48 h)	A375SM, MDA-MB-231, MIA PaCa, and PC-3 cells	E-GNPs can effectively inhibit the nuclear translocation and transcriptional activity of nuclear factor-kappaB (NF-κB) and induce apoptosis in cancer cells.	Chavva et al., 2019
EGCG-radioactive gold (^198^AuNPs) nanoparticles	Reduction of H^198^AuCl_4_·3H_2_O by EGCG	15–40	—	—	PC-3 xenograft SCID mice	The ability of EGCG to target laminin receptor (67LR) leads to the internalization of EGCG-^198^AuNPs into prostate tumor cells, increasing the radiotherapeutic effect of ^198^AuNPs in reducing tumor volumes.	Shukla et al., 2012
EGCG-pNG particles	EGCG and pNG were physically mixed via ultrasonication	50	27	36.2% (36°C, pH 1.2, 0.5 h)	MBT-2 tumor cells and female C3H/He mice	EGCG-pNG mediated tumor apoptosis was demonstrated to involve activation of the caspase cascade, via the Bcl-family proteins, of the mitochondrial pathway.	Hsieh et al., 2012
EGCG-physical nanogold (pNG) particles	EGCG and pNG were physically mixed via ultrasonication	64.7–127.4	29	—	murine B16F10 melanoma cells and C57/BL6 mouse model	The combined EGCG-pNG exerts an improved effect in inhibiting the growth of B16F10 melanoma cells through cell apoptosis.	Chen C. C. et al., 2014
**Mesoporous silica-based EGCG delivery systems**							
Colloidal mesoporous silica (CMS)- EGCG	CMS was dipped in EGCG solution for loading EGCG	50	66	—	HeLa cells	CMS inhibited the collision of EGCG radicals, prolonged the half-life of EGCG, and improved the therapeutic effect of EGCG via inducing cell apoptosis by increasing H_2_O_2_ production.	Ding et al., 2012
CMS@PEGA- pVEC peptide@ EGCG	CMS@peptide was dipped in EGCG solution for loading EGCG	100	—	23% (pH 7.4, 20 h, room temperature	MCF-7 cells and MCF-7 tumor-bearing mice	EGCG induces apoptosis of MCF-7 cancer cells and reduces the change of apoptosis-related proteins with no damage to normal tissue.	Ding et al., 2015
**Lipid-based EGCG delivery systems**							
EGCG loaded solid lipid nanoparticles (EGCG-SLN)	Emulsion-solvent evaporation method	157	67.2	83.9% (37°C, pH 5, 12 h)	MDA-MB 231 and DU-145 cells	EGCG-SLN caused an 8.1-fold increase in cytotoxicity of EGCG against MDA-MB-231 and 3.8 times increase against DU-145.	Radhakrishnan et al., 2016
EGCG-SLN;	High shear homogenization and ultrasonication technique	364 ± 11	83	40% (37°C, pH 1.2, 24 h)	Caco-2 cells	Both SLN and NLC were successfully developed for EGCG protection and stabilization and can be a useful platform for the enhancement of EGCG bioavailability.	Frias et al., 2016
Folic acid-functionalized EGCG-loaded NLC	High shear homogenization and ultrasonication technique	300	90	—	Caco-2 cells	Folic acid functionalization of EGCG-loaded lipid NPs can successfully increase its transport across the intestinal Barrier.	Granja et al., 2019
**Chitosan-based EGCG delivery systems**							
EGCG-loaded chitosan-gellan gum bipolymeric nanohydrogels	Ionotropic gelation and polyelectrolyte complexation technique	250	91.85	53.4% (pH 7.4, 24 h)	P. aeruginosa, *E. coli*, B. subtilis and *S. aureus*	EGCG-loaded nanohydrogels displayed sustained drug release and better antibacterial, antioxidant activity.	Dahiya et al., 2017
Chitosan nanoparticles encapsulating EGCG (Chit-nanoEGCG)	Sonication and dialysis method	150–200	—	10% (simulated gastric juice, 24 h); 50% (simulated intestinal fluid, 24 h)	22Rν1 cells and ahymic nude mice	Chit-nanoEGCG led to sustained release of EGCG, inducing poly (ADP-ribose) polymerases cleavage; increasing protein expression of Bax with concomitant decrease in Bcl-2; activating caspases, reducing Ki-67 and proliferating cell 150 nuclear antigen.	Khan et al., 2013
Folate conjugated chitosan coated EGCG nanoparticles (FCS-EGCG-NPs)	Ionic cross-linking method	400	75	—	HeLa, H1299 and Capan-1 cells	FCS-EGCG-NPs had a greater tumor inhibition effect on cancer cells having a large expression of folic acid receptors on the surface.	Liang et al., 2014
**Protein-based EGCG delivery systems**							
β-lactoglobulin (β-Lg)-EGCG nanoparticles	Thermally-induced protein-EGCG co-assemblies	—	58.6 ± 6.8	25% (37°C, 180 min)	—	The very limited release from β-Lg-EGCG nanoparticles during simulated gastric digestion made it a potential enteric carrier for polyphenols.	Shpigelman et al., 2012
EGCG-β-Lg nanoparticles (Eβ-NPs)	Thermally-induced protein-EGCG co-assemblies	31.3 ± 0.62	59.2	—	FACS. A375 and TE-1 cells	The Eβ-NPs possessed better bioactivity than native EGCG with respect to the proliferative inhibition of cancer cells.	Wu et al., 2017
β-Lg, 3-mercapto-1-hexanol (3MH) and EGCG co-assembled nanocomplexes (MEβ-NPs)	Thermally-Induced protein-EGCG co-assemblies	28.4–32.3	50.2–60.8	62.0% (37°C, 24 h)	A375, Hep G2 and TE-1 cells	Antioxidant capacity, absorbability and bioavailability of EGCG in MEβ-NPs was improved, exhibiting greater stability, sustained release and anticancer effects *in vitro* and *in vivo* than free EGCG.	Yang Y. et al., 2017
EGCG-loaded β-lactoglobulin (BLG)–chlorogenic acid (CA) conjugates	Free radical method	105–110	71.8% [BLG-CA (low)]; 73.5% [BLG-CA (high)]	34.5% (pH 7.4, 6 h)	—	BLG-CA (high) showed higher inhibition of EGCG release than BLG-CA (low), suggesting that CA exhibited inhibition for digestive enzymes in intestinal stage.	Fan et al., 2017
Ferritin-chitosan Maillard reaction products (FCMPs)-EGCG complexes	EGCG solution was dripped into FCMPs solution for EGCG encapsulating	7.5	12.87 (w/w)	75.6% (simulated gastric fluid, 160 min)	Caco-2 monolayer model	The glycosylated ferritin retained its shell-like structure and can protect the encapsulated EGCG in simulated gastrointestinal tract. The ferritin-chitosan double shells can improve the absorption of encapsulated EGCG in Caco-2 monolayer model.	Yang R. et al., 2017

### Gold Nanoparticles-Based EGCG Delivery Systems

AuNPs-enabled drug delivery systems for cancer chemotherapy have been intensively studied due to their high surface to volume ratio and controllable functionalized surface, which are favorable properties in drug loading and delivery. To date, AuNPs-based EGCG delivery systems have been substantially explored in cancer diagnosis and therapy. Previous research revealed that reduction of Au^3+^ by EGCG offered a promising synthetic route of Au nanoparticles with an EGCG-grafted surface (Viator et al., 2010; Shukla et al., 2012; Chavva et al., 2019), which is contributive to improving uptake by tumor cells. Moreover, the radioactive gold source (H^198^AuCl_4_) enables a radiotherapeutic effect of EGCG-^198^AuNPs in killing prostate tumor cells (Shukla et al., 2012). The mixing of EGCG and physical nanogold (pNG) particles is also an effective method for coating EGCG on AuNPs with a prolonged half-life (110 days) (Hsieh et al., 2012). The anti-tumor mechanism of EGCG-pNG is attributed to the mitochondrial pathway-mediated apoptosis, demonstrating a 1.66-fold higher inhibition ratio than free EGCG (Chen C. C. et al., 2014).

### Mesoporous Silica-Based EGCG Delivery Systems

Mesoporous silica nanoparticles (MSNs), with controllable size, ordered porosity, high internal surface area, and easily modified surfaces, have been widely applied for the construction of drug delivery systems. The high corrosion resistance of MSN under physiological conditions provides protection for the encapsulated drugs from degradation before reaching the tumor sites. The high biocompatibility of MSN with negligible cytotoxicity also makes it a safe drug carrier that can be eliminated through renal clearance (Chen et al., 2013; Li et al., 2017). According to a previous study (Ding et al., 2012), MSN can adsorb EGCG through electrostatic attraction and inhibit the collision of EGCG radicals, thus prolonging the half-life of EGCG. Our group further introduced a breast-tumor-homing cell-penetrating peptide onto the EGCG-loaded MSN for the targeted accumulation and release of EGCG in MCF-7 cells (Ding et al., 2015).

### Lipid-Based EGCG Delivery Systems

Nanocapsules with lipids in the matrix could improve their biocompatibility. The high encapsulation efficiency and controlled-release from lipid-based nanovehicles maintain the prolonged efficacy of entrapped drugs. Solid lipid nanoparticles (SLN) consisting of glycerol monostearate, stearic acid, and soya lecithin were used as stealth vehicles for efficient EGCG delivery due to their high biocompatibility, while the erosion or metabolization of lipids led to the sustained release of EGCG (Radhakrishnan et al., 2016). In comparison with SLN, nanostructured lipid carriers (NLC) that consisted of both solid (Precirol® ATO) and liquid (miglyol-812) lipids showed higher encapsulation efficiency (90%) and stability during long-term storage (Frias et al., 2016). Moreover, more than 60% of encapsulated EGCG remains in the nanovehicles after contact with simulated gastric and intestinal fluids for 4 h, indicating the feasibility of EGCG-NLC for oral administration. On this basis, folic acid is introduced to make it easier for NLC-based nanovehicles to transport across the intestinal barrier (Granja et al., 2019).

### Chitosan-Based EGCG Delivery Systems

As mentioned above, chitosan can serve as a surface modification agent to increase cellular uptake and conduct on-demand drug release, due to its biocompatibility and biodegradability. In addition, cross-linking agents or other structural promoters were combined in these dose forms to prevent chitosan capsules from burst breaking in acidic medium and ensure its applicability as drug carriers in oral administration. Gellan gum with high resistance to heat and acidic media was employed to construct chitosan-gellan gum bipolymeric nanohydrogels for oral EGCG delivery (Dahiya et al., 2017). The interaction between -NH_2_ in chitosan and phosphate in pentasodium tripolyphosphate hexahydrate can also enhance the stability of EGCG-entrapped chitosan nanoparticles (Khan et al., 2013). Moreover, folic acid is introduced via the ionic gelation method for targeted EGCG delivery toward cancer cells with overexpressed folic acid receptors (Liang et al., 2014).

### Protein-Based EGCG Delivery Systems

Proteins are effective nanocarriers for drug delivery systems since these renewable biomacromolecules exhibit a high drug-binding capacity with low cytotoxicity, which can also target tumor cells effectively due to the specific recognition of the receptors on tumor cell membranes. Moreover, proteins with a unique structure provide specific binding sites for EGCG, thus further enhancing the stability of encapsulated EGCG in comparison with other nanovehicles (Yang R. et al., 2017). Thermally-induced protein-EGCG co-assembly is a widely used strategy for constructing β-lactoglobulin (β-Lg)-based EGCG delivery systems with encapsulation efficiencies between 50 and 60% (Shpigelman et al., 2012; Wu et al., 2017; Yang Y. et al., 2017). The limited release (25%) of EGCG in acidic medium (pH 2, 37°C, 3 h) demonstrates the protection of β-Lg nanocapsules for EGCG from gastric digestion and oxidative degradation (Shpigelman et al., 2012). Moreover, the intestinal absorption of β-Lg-EGCG nanoparticles (<50 nm), followed by the sustained release of EGCG in neutral physiological conditions, ensures the high bioavailability of EGCG for cancer therapy. Conjugation of β-Lg with chlorogenic acid (CA) is another route to isolate the protein-based nanovehicles from enzymatic digestion, resulting in the limited premature release of EGCG during the delivery process (Fan et al., 2017).

In comparison with inorganic nanovehicles (Au NPs and MSN, etc.), organic nanocarriers based on nanolipids, chitosan, and proteins possess a higher encapsulation efficiency and exhibit the controllable release of EGCG. The superior biocompatibility with an easier-to-functionalize surface also makes it possible for targeted delivery and enhanced intercellular accumulation of EGCG. However, the relatively low stability restricts their application for EGCG delivery through oral administration where burst breaking or biodegradation can occur in digestive fluids, resulting in the pre-release and structural change of entrapped EGCG. Therefore, recent research is mainly focused on exploring biomolecule-based nanoplatforms with well-defined nanostructures and sufficient stability for EGCG delivery.

## Combination Therapy by Egcg-Loaded Nanovehicles

Although a range of EGCG-nanovehicles have contributed to the success in maintaining chemical structure integrity and increasing delivery efficiency, nanovehicle-enabled EGCG delivery for single-drug therapy is still suboptimal due to its inherent defects in pharmacological activities. To upgrade the curative effects of EGCG-involved therapy, it is of great importance to integrate multiple therapeutic approaches actuated by diverse rationales. On the other hand, considering side effects or drug resistance in the medication by conventional chemotherapeutics, gene therapeutics, and photosensitizers, introducing EGCG in the formulation would offer the possibility of boosting the therapeutic efficacy. Nanostructured drug carriers offer a versatile scaffold for the integration of EGCG with other therapeutic agents featuring chemotherapy, gene therapy, and phototherapy in a collaborative manner, rendering combination treatment more effective in curing various cancers. Here, we reviewed the recent progresses made in cancer combination therapy that encompass EGCG and therapeutic agents with different functions, and highlighted the advantages of EGCG-involved co-delivery nanovehicles.

### Combination With Other Chemical Drugs

Multidrug resistance (MDR) is one of the major problems facing cancer chemotherapy due to the expression of energy-dependent drug efflux pumps on the plasma membrane. Moreover, side effects induced by anticancer drugs are another formidable factor that cause damage to normal tissues during cancer treatment. As is already known, EGCG is a promising candidate that can overcome the drawbacks of conventional chemotherapeutic agents and enhance their anticancer capabilities. The integration of EGCG and DOX is currently the most in-depth studied EGCG-involved binary therapeutic system. As reported by Yao et al. (2017), DOX-induced cardiotoxicity can be suppressed by EGCG via upregulating the expression of mitochondrial membrane potential and manganese superoxide dismutase. Moreover, EGCG can also ameliorate DOX-evoked oxidative stress injury and activate the ErbB2-involved pro-survival pathway (Saeed et al., 2015). Remarkably, the efficient interaction between EGCG and DOX leads to the high encapsulation efficiency of DOX (88%) within PEG-EGCG micellar nanocomplexes, improving blood circulation stability and tumor targeting ability of DOX while minimizing its dose-dependent side effects (Liang et al., 2018). In the orchestration of EGCG and DOX, EGCG was reported to inhibit the activity of MMP-2 and MMP-9 that are increased in MCF7/DOX cells, thus sensitizing them to DOX and reducing their metastatic potential (Stearns et al., 2010; Nowakowska and Tarasiuk, 2016). Moreover, the pro-survival autophagy of tumor cells can also be reduced by EGCG through targeting and decreasing autophagy signaling induced by DOX treatment, therefore improving the efficacy of DOX (Chen L. et al., 2014; Wang W. et al., 2018).

### Combination With Gene Therapy

Gene therapy is achieved by counteracting or replacing a malfunctioning gene within the cells, and exhibits great potential to treat various cancers at their genetic roots (Naldini, 2015). In particular, the sequence-specific gene silencing induced by RNA interference (RNAi) can modulate the immune response, regulate the cell cycle, inhibit the overexpressed oncogenes, induce apoptosis of tumor cells, and has anti-angiogenesis effect, which can also amplify the efficacy of chemotherapeutic agents. Recently, the enhanced therapeutic efficacy derived from the integration of EGCG and therapeutic genes has caught great attention. It has been confirmed that 28 genes related to the pro-apoptotic (activate) and pro-survival (inhibit) of Hs578T cells are altered by the combination treatment of EGCG and p53siRNA (Braicu et al., 2015). The integration of specific siRNA and EGCG can also reverse the drug resistance of tumor cells to conventional chemotherapy agents such as tamoxifen (Esmaeili, 2015). Based on investigations at the cellular level, nanovehicles were developed for co-delivering therapeutic genes and EGCG to realize the combined treatment of cancer *in vivo*. As reported by Ding et al. (2018), siRNA and EGCG are self-assembled to form a stealth nanovehicle using protamine as the assembly skeleton, which can accommodate the two therapeutic agents and minimize side effects. In addition, the as-obtained nanovehicles modified with hyaluronic acid and tumor-homing cell-penetrating peptide demonstrated superior selectivity toward drug-resistant MDA-MB-231 cell lines with marked enhancement in cytotoxicity, which is 15 times greater than free EGCG.

### Combination With Phototherapy

Photodynamic therapy (PDT) is an emerging cancer treatment strategy based on the photochemical reactions aroused by photosensitizers, which can produce ROS with high cytotoxicity under irradiation at specific wavelengths, thereby inducing apoptosis of tumor cells and causing damage to tumor tissues. The superposition of EGCG-dominated chemotherapy and PDT in a single nanostructured entity provides a practical approach to enhance cancer therapeutic efficacy. The synergism of PDT (Radachlorin, 662 nm laser) and EGCG via intratumoral injection leads to the increased expression of primary antibodies, such as p21, p53, Bax, and PARP, causing significant enhancement in TC-1 tumor cell growth inhibition compared to PDT or EGCG-involved therapy alone (Mun et al., 2014). In addition, the irradiation of pulsed laser light can in turn contribute to transmembrane convection of EGCG by modulating the nanostructure of water layers in tumor cells (Sommer et al., 2011). In view of the synergism between EGCG and PDT, the photoreponsive nanovehicles are in great demand for EGCG delivery and release. However, related research is still rare. Notably, DOX-loaded EGCG-Fe(III) networks have been demonstrated to realize chemo- and photothermal therapy (PTT) simultaneously (Chen et al., 2019). Specifically, the photothermal capability of EGCG-Fe(III) networks under near-infrared irradiation cause damage to HT-29 cells through hyperthermia, thus reinforcing the chemotherapy efficacy of DOX in inducing tumor cell apoptosis and eventually ablating the solid tumor completely. Anisotropic gold nanostructures with fascinating photothermal properties can act as nanocarriers for delivering therapeutic molecules, but their uses in EGCG loading for chemo-photothermal combination therapy are still seldom reported. The recruitment of anisotropic gold nanostructures is a promising complement to EGCG for improving cancer therapy efficacy.

## Conclusions

This review summarizes the achievements of various popular nanovehicles for EGCG delivery *in vitro* and *in vivo*. The recent progresses showed that nanovehicles designed with structural characteristics and surface functionalities allowed targeted delivery and controllable release of EGCG with enhanced stability and bioavailability. However, clinical applications of these EGCG-containing nanoplatforms are still limited. The chemically stable AuNPs and MSNs suffered severe accumulation in the liver and spleen, potentially causing toxicity to the human body. Meanwhile, the biodegradation of organic nanoparticles (e.g., nanolipid-based, chitosan-based, and protein-based nanoparticles) in digestive fluids may restrict their application for EGCG delivery through oral administration. Therefore, it is essential and urgent to develop nanocarriers with considerable stability, biocompatibility, efficiency, and safety, which would be adaptive to clinical practices of EGCG-involved therapy in the future. To this end, more attention should be paid to non-toxic and biodegradable nanomaterials with high internal surface areas, such as layered double hydroxides (LDHs), blank phosphorus (BP), and metal organic frameworks (MOFs), although studies on them for EGCG delivery are still lacking.

In comparison with the single-drug delivery systems, integration of EGCG with other therapeutic agents enabling chemotherapy, gene therapy, or phototherapy to form multifunctional nanoplatforms appears to be an ideal strategy in enhancing the efficacy of cancer treatment. Moreover, fully understanding the working principles of EGCG and other chemo-, gene, and phototherapeutic agents in cancer cells offer theoretical evidence supporting combination therapy, according to which arrangement and gathering of therapeutic agents in one nanostructure can be rationalized to maximize the potency and minimize the undesired effects. Guided by the mechanisms of synergistic action in combination therapy, we can envision that interfaces between EGCG chemotherapy and other emerging therapeutic modalities including starvation therapy, gas therapy, chemodynamic therapy, and immunity therapy would be further explored and engineered to formulate comprehensive and effective combination treatments of cancer.

## Author Contributions

KL designed and wrote the review with input from CT and QM for conceiving, writing, and editing the manuscript. All authors contributed to the article and approved the submitted version.

## Conflict of Interest

The authors declare that the research was conducted in the absence of any commercial or financial relationships that could be construed as a potential conflict of interest.

## References

[B1] Berletch J. B. Liu C. Love W. K. Andrews L. G. Katiyar S. K. Tollefsbol T. O. (2008). Epigenetic and genetic mechanisms contribute to telomerase inhibition by EGCG. J. Cell. Biochem. 103, 509–519. 10.1002/jcb.21417 17570133 PMC2435482

[B2] Braicu C. Pileczki V. Pop L. Petric R. C. Chira S. Pointiere E. (2015). Dual targeted therapy with p53 siRNA and epigallocatechingallate in a triple negative breast cancer cell model. PLoS ONE 10:e0120936. 10.1371/journal.pone.0120936 25849487 PMC4388814

[B3] Cai Y. Z. Mei S. Jie X. Luo Q. Corke H. (2006). Structure-radical scavenging activity relationships of phenolic compounds from traditional ChinesChakrabartie medicinal plants. Life Sci. 78, 2872–2888. 10.1016/j.lfs.2005.11.004 16325868

[B4] Chakrawarti L. Agrawal R. Dang S. Gupta S. Gabrani R. (2016). Therapeutic effects of EGCG: a patent review. Expert Opin. Ther. Pat. 26, 907–916. 10.1080/13543776.2016.1203419 27338088

[B5] Chavva S. Deshmukh S. Kanchanapally R. Tyagi N. Coym J. Singh A.. (2019). Epigallocatechin gallate-gold nanoparticles exhibit superior antitumor activity compared to conventional gold nanoparticles: potential synergistic interactions. Nanomaterials 9:396. 10.3390/nano9030396 30857226 PMC6474148

[B6] Chen C. C. Hsieh D. S. Huang K. J. Chan Y. L. Hong P. D. Yeh M. K.. (2014). Improving anticancer efficacy of (–)-epigallocatechin-3-gallate gold nanoparticles in murine B16F10 melanoma cells. Drug Des. Dev. Ther. 8, 459–474. 10.2147/DDDT.S58414 24855338 PMC4020885

[B7] Chen L. Ye H. L. Zhang G. Yao W. M. Chen X. Z. Zhang F. C.. (2014). Autophagy inhibition contributes to the synergistic interaction between EGCG and Doxorubicin to kill the hepatoma Hep3B cells. PLoS ONE 9:e85771. 10.1371/journal.pone.0085771 24465696 PMC3897495

[B8] Chen X. Yi Z. Chen G. Ma X. Su W. Cui X. (2019). DOX-assisted functionalization of green tea polyphenol nanoparticles for effective chemo-photothermal cancer therapy. J. Mater. Chem. B 7, 4066–4078. 10.1039/C9TB00751B

[B9] Chen Y. Chen H. Shi J. (2013). *In vivo* bio-safety evaluations and diagnostic/therapeutic applications of chemically designed mesoporous silica nanoparticles. Adv. Mater. 25, 3144–3176. 10.1002/adma.201205292 23681931

[B10] Cragg G. M. Grothaus P. G. Newman D. J. (2009). Impact of natural products on developing new anti-cancer agents. Chem. Rev. 109, 3012–3043. 10.1021/cr900019j 19422222

[B11] Dahiya S. Rani R. Kumar S. Dhingra D. Dilbaghi N. (2017). Chitosan-gellan gum bipolymeric nanohydrogels-a potential nanocarrier for the delivery of epigallocatechin gallate. BioNanoSci 7, 508–520. 10.1007/s12668-017-0416-0

[B12] Ding J. Kong X. Yao J. Wang J. Cheng X. Tang B. (2012). Core-shell mesoporous silica nanoparticles improve HeLa cell growth and proliferation inhibition by (–)-epigallocatechin-3-gallate by prolonging the half-life. J. Mater. Chem. 22, 19926–19931. 10.1039/c2jm32271d

[B13] Ding J. Liang T. Min Q. Jiang L. Zhu J. J. (2018). “Stealth and fully-laden” drug carriers: Self-assembled nanogels encapsulated with epigallocatechin gallate and siRNA for drug-resistant breast cancer therapy. ACS Appl. Mater. Interfaces 10, 9938–9948. 10.1021/acsami.7b19577 29436217

[B14] Ding J. Yao J. Xue J. Li R. Bao B. Jiang L.. (2015). Tumor-homing cell-penetrating peptide linked to colloidal mesoporous silica encapsulated (–)-epigallocatechin-3-gallate as drug delivery system for breast cancer therapy *in vivo*. ACS Appl. Mater. Interfaces 7, 18145–18155. 10.1021/acsami.5b05618 26225796

[B15] Esmaeili M. A. (2015). Combination of siRNA-directed gene silencing with epigallocatechin-3-gallate (EGCG) reverses drug resistance in human breast cancer cells. J. Chem. Biol. 9, 41–52. 10.1007/s12154-015-0144-2 26855680 PMC4733072

[B16] Fan Y. Zhang Y. Yokoyama W. Yi J. (2017). β-Lactoglobulin–chlorogenic acid conjugate-based nanoparticles for delivery of (–)-epigallocatechin-3-gallate. RSC Adv. 7, 21366–21374. 10.1039/C6RA28462K

[B17] Frias I. Neves A. Pinheiro M. Reis S. (2016). Design, development, and characterization of lipid nanocarriers-based epigallocatechin gallate delivery system for preventive and therapeutic supplementation. Drug Des. Dev. Ther. 10, 3519–3528. 10.2147/DDDT.S109589 27826184 PMC5096752

[B18] Gan R. Y. Li H. B. Sui Z. Q. Corke H. (2017). Absorption, metabolism, anti-cancer effect and molecular targets of epigallocatechin gallate (EGCG): an updated review. Crit. Rev. Food Sci. Nutr. 58, 924–941. 10.1080/10408398.2016.1231168 27645804

[B19] Granja A. Neves A. R. Sousa C. T. Pinheiro M. Reis S. (2019). EGCG intestinal absorption and oral bioavailability enhancement using folic acid-functionalized nanostructured lipid carriers. Heliyon 5:e02020. 10.1016/j.heliyon.2019.e02020 31317081 PMC6611934

[B20] Granja A. Pinheiro M. Reis S. (2016). Epigallocatechin gallate nanodelivery systems for cancer therapy. Nutrients 8:307. 10.3390/nu8050307 27213442 PMC4882719

[B21] Hsieh D. S. Lu H. C. Chen C. C. Wu C. J. Yeh M. K. (2012). The preparation and characterization of gold-conjugated polyphenol nanoparticles as a novel delivery system. Int. J. Nanomed. 7, 1623–1633. 10.2147/IJN.S30060 22615529 PMC3357049

[B22] Hu F. Wei F. Wang Y. Wu B. Fang Y. Xiong B. (2015). EGCG synergizes the therapeutic effect of cisplatin and oxaliplatin through autophagic pathway in human colorectal cancer cells. J. Pharmacol. Sci. 128, 27–34. 10.1016/j.jphs.2015.04.003 26003085

[B23] Khan N. Bharali D. J. Adhami V. M. Siddiqui I. A. Cui H. Shabana S. M.. (2013). Oral administration of naturally occurring chitosan-based nanoformulated green tea polyphenol EGCG effectively inhibits prostate cancer cell growth in a xenograft model. Carcinogenesis 35, 415–423. 10.1093/carcin/bgt321 24072771 PMC3908746

[B24] Li G. X. Chen Y. K. Hou Z. Xiao H. Jin H. Lu G.. (2010). Pro-oxidative activities and dose-response relationship of (–)-epigallocatechin-3-gallate in the inhibition of lung cancer cell growth: a comparative study *in vivo* and *in vitro*. Carcinogenesis 31, 902–910. 10.1093/carcin/bgq039 20159951 PMC2864413

[B25] Li Y. Li N. Pan W. Yu Z. Yang L. Tang B. (2017). Hollow mesoporous silica nanoparticles with tunable structures for controlled drug delivery. ACS Appl. Mater. Interfaces 9, 2123–2129. 10.1021/acsami.6b13876 28004570

[B26] Liang J. Cao L. Zhang L. Wan X. C. (2014). Preparation, characterization, and *in vitro* antitumor activity of folate conjugated chitosan coated EGCG nanoparticles. Food Sci. Biotechnol. 23, 569–575. 10.1007/s10068-014-0078-4

[B27] Liang T. Yao Z. Ding J. Min Q. Jiang L. P. Zhu J. J. (2018). Cascaded aptamers-governed multistage drug delivery system based on biodegradable envelope type nanovehicle for targeted therapy of HER2-overexpressing breast cancer. ACS Appl. Mater. Interfaces. 10, 34050–9. 10.1021/acsami.8b14009 30207689

[B28] Mignani S. Rodrigues J. Tomas H. Zablocka M. Shi X. Caminade A. M.. (2018). Dendrimers in combination with natural products and analogues as anti-cancer agents. Chem. Soc. Rev. 47, 514–532. 10.1039/C7CS00550D 29154385

[B29] Mun S. T. Bae D. H. Ahn W. S. (2014). Epigallocatechin gallate with photodynamic therapy enhances anti-tumor effects *in vivo* and *in vitro*. Photodiagn. Photodyn. Ther. 11, 141–147. 10.1016/j.pdpdt.2014.03.003 24632332

[B30] Nakagawa K. Miyazawa T. (1997). Chemiluminescence-high-performance liquid chromatographic determination of tea catechin, (–)-epigallocatechin 3-gallate, at picomole levels in rat and human plasma. Anal. Biochem. 248, 41–49. 10.1006/abio.1997.2098 9177723

[B31] Naldini L. (2015). Gene therapy returns to centre stage. Nature 526, 351–360. 10.1038/nature15818 26469046

[B32] Nowakowska A. Tarasiuk J. (2016). Comparative effects of selected plant polyphenols, gallic acid and epigallocatechin gallate, on matrix metalloproteinases activity in multidrug resistant MCF7/DOX breast cancer cells. Acta Biochim. Pol. 63, 571–575. 10.18388/abp.2016_1256 27231728

[B33] Qi H. Abe N. Zhu B. Murata Y. Nakamura Y. (2014). (–)-Epigallocatechin-3-gallate ameliorates photodynamic therapy responses in an *in vitro* T lymphocyte model. Phytother. Res. 28, 1486–1491. 10.1002/ptr.5152 24700514

[B34] Radhakrishnan R. Kulhari H. Pooja D. Gudem S. Bhargava S. Shukla R.. (2016). Encapsulation of biophenolic phytochemical EGCG within lipid nanoparticles enhances its stability and cytotoxicity against cancer. Chem. Phys. Lipids 198, 51–60. 10.1016/j.chemphyslip.2016.05.006 27234272

[B35] Sadava D. Whitlock E. Kane S. E. (2007). The green tea polyphenol, epigallocatechin-3-gallate inhibits telomerase and induces apoptosis in drug-resistant lung cancer cells. Biochem. Biophys. Res. Commun. 360, 233–237. 10.1016/j.bbrc.2007.06.030 17585882

[B36] Saeed N. M. El-Naga R. N. El-Bakly W. M. Abdel-Rahman H. M. El-Demerdash E. (2015). Epigallocatechin-3-gallate pretreatment attenuates doxorubicin-induced cardiotoxicity in rats: a mechanistic study. Biochem. Pharmacol. 95, 145–155. 10.1016/j.bcp.2015.02.006 25701654

[B37] Shankar S. Marsh L. Srivastava R. K. (2012). EGCG inhibits growth of human pancreatic tumors orthotopically implanted in Balb C nude mice through modulation of FKHRL1/FOXO3a and neuropilin. Mol. Cell. Biochem. 372, 83–94. 10.1007/s11010-012-1448-y 22971992 PMC3508371

[B38] Shpigelman A. Cohen Y. Livney Y. D. (2012). Thermally-induced β-lactoglobuline-EGCG nanovehicles: loading, stability, sensory and digestive-release study. Food Hydrocolloids 29, 57–67. 10.1016/j.foodhyd.2012.01.016

[B39] Shukla R. Chanda N. Zambre A. Upendran A. Katti K. Kulkarni R. R.. (2012). Laminin receptor specific therapeutic gold nanoparticles (^198^AuNP-EGCg) show efficacy in treating prostate cancer. Proc. Natl. Acad. Sci. U.S.A. 109, 12426–12431. 10.1073/pnas.1121174109 22802668 PMC3411993

[B40] Singh B. N. Shankar S. Srivastava R. K. (2011). Green tea catechin, epigallocatechin-3-gallate (EGCG): Mechanisms, perspectives and clinical applications. Biochem. Pharmacol. 82, 1807–1821. 10.1016/j.bcp.2011.07.093 21827739 PMC4082721

[B41] Singh T. Katiyar S. K. (2013). Green tea polyphenol, (–)-epigallocatechin-3-gallate, induces toxicity in human skin cancer cells by targeting beta-catenin signaling. Toxicol. Appl. Pharmacol. 273, 418–424. 10.1016/j.taap.2013.09.021 24096034 PMC3884646

[B42] Sommer A. P. Zhu D. Mester A. R. Försterling H. D. (2011). Pulsed laser light forces cancer cells to absorb anticancer drugs-The role of water in nanomedicine. Artif. Cells Blood Substit. Biotechnol. 39, 169–173. 10.3109/10731199.2010.516262 20849242

[B43] Spradlin J. N. Hu X. Ward C. C. Brittain S. M. Jones M. D. Ou L.. (2019). Harnessing the anti-cancer natural product nimbolide for targeted protein degradation. Nat. Chem. Biol. 15, 747–755. 10.1038/s41589-019-0304-8 31209351 PMC6592714

[B44] Stearns M. E. Amatangelo M. D. Varma D. Sell C. Goodyear S. M. (2010). Combination therapy with epigallocatechin-3-gallate and Doxorubicin in human prostate tumor modeling studies. Am. J. Pathol. 177, 3169–3179. 10.2353/ajpath.2010.100330 20971741 PMC2993277

[B45] Viator J. A. Gupta S. Goldschmidt B. S. Bhattacharyya K. Kannan R. Shukla R.. (2010). Gold nanoparticle mediated detection of prostate cancer cells using photoacoustic flowmetry with optical reflectance. J. Biomed. Nanotechnol. 6, 187–191. 10.1166/jbn.2010.1105 20738074

[B46] Wang J. Man G. C. W. Chan T. H. Kwong J. Wang C. C. (2018). A prodrug of green tea polyphenol (–)-epigallocatechin-3-gallate (Pro-EGCG) serves as a novel angiogenesis inhibitor in endometrial cancer. Cancer Lett. 412, 10–20. 10.1016/j.canlet.2017.09.054 29024813

[B47] Wang W. Chen D. Zhu K. (2018). SOX2OT variant 7 contributes to the synergistic interaction between EGCG and Doxorubicin to kill osteosarcoma via autophagy and stemness inhibition. J. Exp. Clin. Cancer Res. 37:37. 10.1186/s13046-018-0689-3 29475441 PMC6389193

[B48] Wu M. Jin J. Jin P. Xu Y. Yin J. Qin D. (2017). Epigallocatechin gallate-β-lactoglobulin nanoparticles improve the antitumor activity of EGCG for inducing cancer cell apoptosis. J. Funct. Foods 39, 257–263. 10.1016/j.jff.2017.10.038

[B49] Xia P. Xu X. Y. (2015). PI3K/Akt/mTOR signaling pathway in cancer stem cells: From basic research to clinical application. Am. J. Cancer Res. 5, 1602–1609. 26175931 PMC4497429

[B50] Yang Q. Q. Wei X. L. Fang Y. P. Gan R. Y. Wang M. Ge Y. Y.. (2019). Nanochemoprevention with therapeutic benefits: an updated review focused on epigallocatechin gallate delivery. Crit. Rev. Food Sci. Nutr. 60, 1243–1264. 10.1080/10408398.2019.1565490 30799648

[B51] Yang R. Liu Y. Gao Y. Wang Y. Blanchard C. Zhou Z. (2017). Ferritin glycosylated by chitosan as a novel EGCG nano-carrier: structure, stability, and absorption analysis. Int. J. Biol. Macromol. 105, 252–261. 10.1016/j.ijbiomac.2017.07.040 28693994

[B52] Yang Y. Jin P. Zhang X. Ravichandran N. Ying H. Yu C. (2017). New epigallocatechin gallate (EGCG) nanocomplexes co-assembled with 3-mercapto-1-hexanol and β-lactoglobulin for improvement of antitumor activity. J. Biomed. Nanotechnol. 13, 805–814. 10.1166/jbn.2017.2400

[B53] Yao Y. F. Liu X. Li W. J. Shi Z. W. Yan Y. X. Wang L. F.. (2017). (–)-Epigallocatechin-3-gallate alleviates doxorubicin-induced cardiotoxicity in sarcoma 180 tumor-bearing mice. Life Sci. 180, 151–159. 10.1016/j.lfs.2016.12.004 27956351

[B54] Ye J. H. Augustin M. A. (2018). Nano- and micro-particles for delivery of catechins: Physical and biological performance. Crit. Rev. Food Sci. Nutr. 59, 1563–1579. 10.1080/10408398.2017.1422110 29345975

[B55] Zhou Y. Tang J. Du Y. Ding J. Liu J. Y. (2016). The green tea polyphenol EGCG potentiates the antiproliferative activity of sunitinib in human cancer cells. Tumor Biol. 37, 8555–8566. 10.1007/s13277-015-4719-x 26733173

[B56] Zhu J. Jiang Y. Yang X. Wang S. Xie C. Li X.. (2017). Wnt/β-catenin pathway mediates (–)-Epigallocatechin-3-gallate (EGCG) inhibition of lung cancer stem cells. Biochem. Biophys. Res. Commun. 482, 15–21. 10.1016/j.bbrc.2016.11.038 27836540

